# Constitutive and Regulatory Responses of *Arabidopsis thaliana* to Harmonically Oscillating Light

**DOI:** 10.1111/ppl.70421

**Published:** 2025-07-31

**Authors:** Yuxi Niu, David Fuente, Shizue Matsubara, Dušan Lazár, Ladislav Nedbal

**Affiliations:** ^1^ Institute of Bio‐ and Geosciences/Plant Sciences (IBG‐2) Jülich Germany; ^2^ Department of Biophysics Wageningen University and Research Wageningen the Netherlands; ^3^ Department of Biophysics, Faculty of Science Palacký University Olomouc Czech Republic

**Keywords:** chlorophyll fluorescence, frequency domain, harmonics, mathematical model, photosynthesis

## Abstract

The rate of net CO_2_ uptake is proportional to dim light and saturates when the light exceeds the plant's assimilation capacity. This simple relationship between constant light and photosynthesis becomes intriguingly complex when the light oscillates. The rates of photosynthesis may differ between the descending and ascending phases of light oscillation. This hysteresis changes with the frequency and amplitude of the light and reports on the dynamics of the photosynthetic reactions and their regulation. Here, we investigated the chlorophyll fluorescence response of *Arabidopsis thaliana* to light oscillating with three different amplitudes: 100–200, 100–400, and 100–800 μmol photons m^−2^ s^−1^, each with periods ranging from 1 s to 8 min. The light amplitudes and periods were chosen to represent light patterns often appearing in nature. Three genotypes were compared: wild‐type Col‐0 and *npq1* and *npq4* mutants that are incapacitated in the rapidly reversible energy‐dependent non‐photochemical quenching (qE). The experiments identified two major dynamic patterns. One was found in oscillation periods shorter than 30 s, characterized by constitutive hysteresis and non‐linearity. The other was mainly formed by regulatory hysteresis, occurring when the oscillation periods were longer than 30 s. The mathematical model simulating the chlorophyll fluorescence dynamics qualitatively reproduced the constitutive and regulatory dynamic patterns observed in the experiments. The model simulations illustrated the dynamics of plastoquinone pool reduction and variables affecting non‐photochemical quenching that form the constitutive and regulatory hysteresis types. The model simulations provided mechanistic insights into molecular processes forming the plant response to oscillating light.

## 1 Introduction

The stimulus–response^1^ relationship is a fundamental concept in biology, characterizing the extent to which an organism responds to the strength, duration, or dose of a stimulus (Calabrese and Baldwin 2003; Mattson 2008; Pinheiro and Duffull 2009). In plant research, this concept is commonly applied to assess optimal growth conditions or plant resistance to stress (Berry and Bjorkman 1980; Idso and Idso 1994; Lee et al. 2007; Dusenge et al. 2019). Another widely used stimulus–response relationship is the photosynthetic light response curve (P–I curve), which describes how the net carbon assimilation rate, P, depends on the intensity of the photosynthetically active radiation, PAR or I (Evans et al. 1993; Ralph and Gademann 2005; Hogewoning et al. 2010; Flood et al. 2011).

A typical P–I curve exhibits three distinct phases. Under low light intensities, the rate of photosynthesis is primarily limited by the availability of light, and therefore, it increases as a linear function of light intensity (Kiss et al. 2008; Krah and Logan 2010; Murchie and Niyogi 2011; Hasan and Cramer 2012). Under high light, photosynthesis is limited by the electron transport and the capacity of the Calvin‐Benson‐Bassham cycle (Murchie and Niyogi 2011; Hasan and Cramer 2012; Hoh et al. 2023) and therefore, it increases little when PAR is increased. Third, the absorbed light energy that exceeds the assimilation capacity of the plant can cause photodamage and, ultimately, photoinhibition that is manifested by decreasing photosynthesis in increasing light (Krause 1988; Aro et al. 1993; Allahverdiyeva and Aro 2012).

The P–I curve of photosynthesis can be determined by measuring steady‐state rates of net CO_2_ uptake or net O_2_ evolution in different light intensities (Evans et al. 1993; Ögren and Evans 1993). An alternative way of estimating the rate of photosynthesis in relation to light intensity is by measuring the relative electron transport rate (ETR) of photosystem II (PSII). The ETR is calculated from the chlorophyll fluorescence measured by the pulse amplitude modulation (PAM) technique that probes the actinic effects of the applied actinic light, combined with saturation pulses of light that transiently close PSII reaction centers (Schreiber 2004).

The P–I curve, measured through O_2_, CO_2_, or chlorophyll fluorescence, characterizes a steady‐state photosynthesis response to constant light exposure. Such a P–I curve represents a fundamental plant stimulus–response, which, however, cannot be used to understand the plant behavior in rapidly changing light that often occurs in nature (Way and Pearcy 2012; Smith and Berry 2013; Kaiser et al. 2018). The light fluctuations in different environments can be roughly classified by their typical frequencies and amplitudes (Table 1). This is, however, only a crude characterization, and there is an endless number of light fluctuation patterns that occur in nature, each pattern potentially leading to different plant responses. On a trivial level, this variability can be illustrated by plant responses to diurnal light modulation in a square, on–off form compared with light modulation that gradually increases from morning to noon and decreases toward the evening, as, for example, in Fondy et al. (1989). The plant responses are different, although the period, duty cycle, and total photon energy per day may be the same in both regimes. Plants will also respond differently when light is modulated by a sine function or by a square wave in minutes, seconds, or shorter. The sine harmonic modulation is analogous to monochromatic light in spectroscopy or a pure musical tone. Square, triangle, or other periodic light modulation patterns are analogous to polychromatic light or complex sounds because they consist of multiple harmonics represented by multiple sine functions. This originates from the uniqueness of harmonic functions of sine or cosine among all other periodic stimulus patterns (Williams 1973; Nuij et al. 2006). No other modulation pattern can be used to analyze complex periodic or even fluctuating pseudo‐periodic light with the clarity of harmonic functions of sine or cosine^2^. Therefore, the stimulation of plants by harmonically modulated light is a unique probe of plant response to a particular frequency and amplitude. In this study, we used harmonically modulated light that was a sine function with periods 1 s ≤ *T* ≤ 8 min, that is, of frequencies 1 Hz ≥ *f* ≥ 2.1 × 10^−3^ Hz. The respective frequencies dominate natural fluctuating light patterns represented in the middle column of Table 1.

**TABLE 1:** The phenomena that result in fluctuations in photosynthetically active radiation (PAR) with different characteristic periods *T* and frequencies *f* = 1/*T* in vegetation canopies.

Period, *T*	1 s > *T* ≥ 10 ms	8 min > *T* ≥ 1 s	*T* ≥ 8 min
Frequency, *f*	100 Hz > *f* ≥ 1 Hz	1 Hz > *f* ≥ 2.1 × 10‐3 Hz	2.1 × 10^−3^ Hz > *f*
Fluctuations against a low light background in vegetation understory	Leaf flutter (Roden and Pearcy 1993) results in very brief sun flecks (Smith and Berry 2013).	Plant swaying (Pearcy et al. 1996) and transient gaps in the canopy (Chazdon and Pearcy 1991) cause sun flecks (Pearcy et al. 1996).	Earth rotation (Pearcy et al. 1996) and canopy gaps (Smith and Berry 2013) result in long‐duration sun patches (Smith and Berry 2013).
Fluctuations against a medium‐range background occurring within canopies	Leaf flutter (Roden and Pearcy 1993) or plant swaying (de Langre 2008) result in brief sun flecks (Pearcy 1990).	Transient gaps in the upper canopy (Chazdon and Pearcy 1991), wind‐induced canopy movement (Peressotti et al. 2001), and intermittent cloudiness (Knapp and Smith 1987) give rise to complex light patterns within canopies (Way and Pearcy 2012; Kaiser et al. 2018).	Earth rotation, canopy gaps, and cloud movement result in sun and cloud patches (Smith and Berry 2013). Diurnal changes create regular irradiance changes (Chazdon and Pearcy 1991)
Fluctuations against a high‐light background in outer canopies	Leaf flutter in canopies creates high‐intensity sun flecks and shade flecks (Pearcy et al. 1996).	Effects of plant swaying (de Langre 2008) and intermittent cloudiness (Knapp and Smith 1987) create sun and cloud flecks (Morales and Kaiser 2020).	Slowly variable irradiance under overcast skies (Navrátil et al. 2007), cloud movement, and diurnal changes (Morales and Kaiser 2020).

The dynamics of photosynthesis under harmonically oscillating light were rarely studied in the past. The pioneering work of Lam and Bungay (1986) and the position paper of Lam et al. (1986) went largely unnoticed. Also, the independently developed line of research using harmonically modulated light (Nedbal and Březina 2002; Nedbal et al. 2003, 2005; Matous et al. 2006; Berger et al. 2007) was seldom cited. Recently, the dynamics of photosynthesis in oscillating light and sensing in the frequency domain have become subjects of renewed interest (Shimakawa and Miyake 2018; Samson et al. 2019; Jose 2021; Nedbal and Lazár 2021; Lazár et al. 2022; Niu et al. 2023, 2024). Lately, a mathematical model has been developed specifically to support the interpretation of decisive mechanisms for the stimulus–response dynamics in oscillating light (Fuente et al. 2024). The model correctly predicted the dispersion, that is, the frequency dependence of the measurable reporter signals, such as the relative chlorophyll fluorescence yield for small amplitudes of light oscillations. However, the model predictions have not yet been confronted with experiments in which large light oscillation amplitudes reach saturation in the P–I curves. Such amplitudes often occur in nature (Table 1) and represent a relevant scenario to study.

This led us to investigate the dependence of the photosynthetic responses to light that oscillated in a broad range of intensities from sub‐saturating to saturating levels. We report on the normalized chlorophyll fluorescence yield, further ChlF(*t*) response of *Arabidopsis thaliana* wild‐type (WT) Columbia (Col‐0), and its *npq1* (Niyogi et al. 1998) and *npq4* mutants (Li et al. 2000) to light that harmonically oscillates between 100 and 200, 100 and 400, and 100 and 800 μmol photons m^−2^ s^−1^ with periods ranging between 1 s and 8 min. The results are presented in the stimulus–response form by plotting ChlF(*t*) against the dynamically changing light intensity, as in Nedbal et al. (2005). Also, the formal concepts of constitutive and regulatory non‐linearity (Bich et al. 2016) were already cited in connection with photosynthesis by Nedbal and Lazár (2021). These approaches are further developed here to classify response dynamics of photosynthesis as constitutive and regulatory hysteresis.

As argued above, the dynamic responses of photosynthesis to harmonically oscillating light of a large amplitude differ from those observed during transients from darkness to light or reverse that are often used in the laboratory to probe, for example, the activation or relaxation of the non‐photochemical quenching (Nilkens et al. 2010; Kress and Jahns 2017). Photosynthesis responds differently to abrupt increases and decreases in light intensity, with forward and reverse reactions happening with different rate constants. For example, the conversion of zeaxanthin to violaxanthin during NPQ relaxation is catalyzed by zeaxanthin epoxidase, whose rate constant is smaller than that of violaxanthin de‐epoxidase (VDE), catalyzing the reverse reactions to convert violaxanthin to zeaxanthin during the NPQ activation (Niyogi et al. 1997; Nilkens et al. 2010; Jahns and Holzwarth 2012; Kress and Jahns 2017). While constant‐light induction and dark relaxation measurements can be used to characterize specific reactions that predominate in one of these two phases, they cannot capture the dynamic responses of photosynthesis to fluctuating light. Harmonically oscillating light provides a framework to study systemic responses in both directions as a function of frequency, offering valuable information on photosynthesis dynamics.

The experimental results were further compared with the simulations obtained by a mathematical model. The original model (Fuente et al. 2024) was modified here to simulate the ChlF(*t*) data obtained in our PAM experiments. This modified model, further called BDM2, reproduced the essential features of the experimental data and explained some of them mechanistically. The residual discrepancy between the experiment and the model simulations is used here to identify knowledge gaps, better understand the regulation of photosynthesis and operational modes in a dynamic light environment, and pave the way for future systems identification.

## 2 Materials and Methods

### 2.1 Experimental Setups

Three genotypes of *A. thaliana* were grown in a climate chamber under light intensity of approximately 100 μmol photons m^˗2^ s^˗1^, including wild‐type Col‐0, the *npq1* mutant that cannot convert violaxanthin into zeaxanthin (Niyogi et al. 1998), and the *npq4* mutant that is deficient in the PsbS protein (Li et al. 2000). Plants were cultivated under controlled environmental conditions, with a 12‐h light/12‐h dark diurnal regime and a day/night air temperature of 26°C/20°C. Relative air humidity was maintained at 60%. Measurements were done between 38 and 43 days after sowing.

The Dual‐KLAS‐NIR spectrophotometer with a 3010‐DUAL leaf cuvette (Heinz Walz GmbH) was used to measure the instantaneous relative chlorophyll fluorescence yield F′(*t*) responding to the actinic light oscillations (Klughammer and Schreiber 2016; Schreiber and Klughammer 2016). The data were collected every 5 ms, and 20 points were averaged to improve the signal‐to‐noise ratio. The resulting time resolution of 0.1 s was sufficient to capture the plant response in light oscillating with a 1 Hz frequency or slower. Red actinic light (630 nm) was applied to both the abaxial and adaxial sides of the leaf. The measuring light was green (540 nm) with an intensity of 6 μmol photons m^−2^ s^−1^, and it was applied only to the abaxial side of the leaf. The plants were collected from the climate chamber before the end of the dark photoperiod and kept in darkness until measurement. Before the oscillating light measurements, each dark‐adapted plant was exposed for 10 min to constant red actinic light (630 nm) to induce photosynthesis. The intensity of this constant light was set to the average of the oscillating light that followed. Thus, plants later exposed to light oscillating between 100 and 200 μmol photons m^−2^ s^−1^ were first acclimated to a constant light intensity of 150 μmol photons m^−2^ s^−1^. Those exposed to oscillations ranging from 100 to 400 μmol photons m^−2^ s^−1^ were initially subjected to a constant light of 250 μmol photons m^−2^ s^−1^ and, similarly, a constant light intensity of 450 μmol photons m^−2^ s^−1^ was used for plants that were later exposed to oscillations between 100 and 800 μmol photons m^−2^ s^−1^.

**FIGURE 1:**
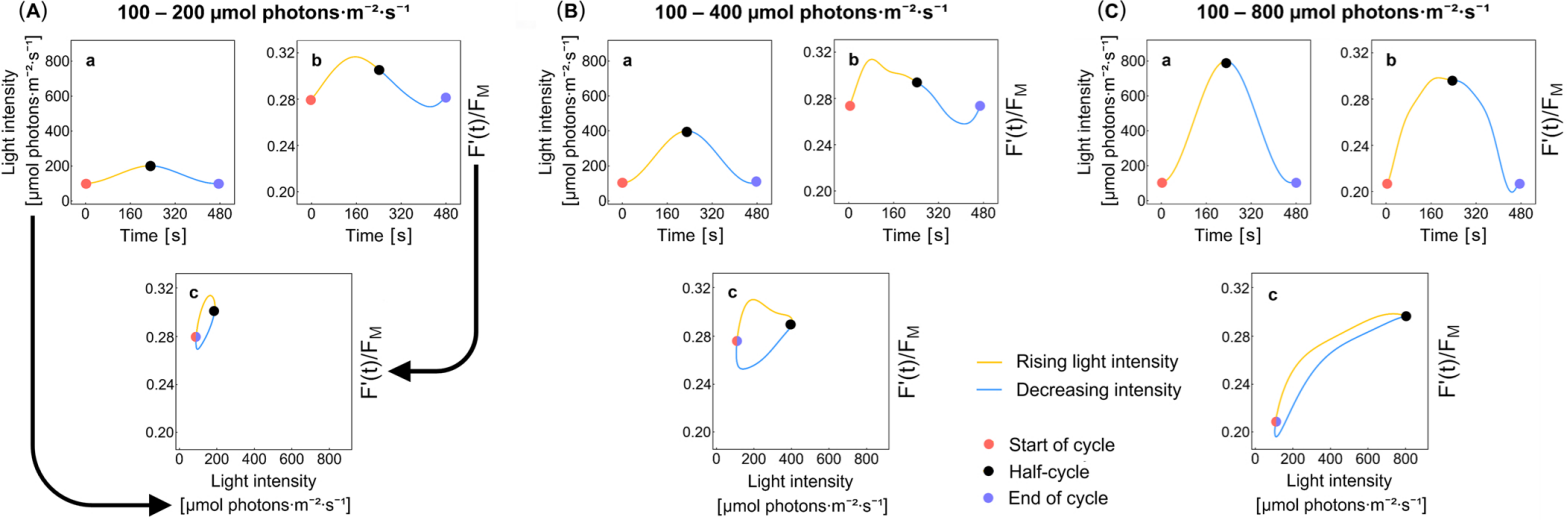
Examples of the normalized ChlF(*t*) = $\frac{F^{\prime }\left(t\right)}{F_{M}}$ of the WT strain Col‐0 to light oscillating with a period *T* = 8 min at three different amplitudes are shown (A–C). The oscillation ranges were 100–200, 100–400, and 100–800 μmol photons m^−2^ s^−1^. The light oscillation (Aa, Ba, Ca) starts at its minimum (red circle), continues with the ascending phase marked by the yellow line to the maximum (black circle), and concludes the period by its descending phase along the blue line to the following minimum (purple circle). The normalized ChlF(*t*) response is shown with the same marking in panels Ab, Bb, and Cb. The same data are shown in the stimulus–response format in Ac, Bc, and Cc, where the line and marker colors are the same as in the a and b panels. The overlapping light minima are marked by half‐purple, half‐red circles in c.

Following the induction in constant actinic light, plants were exposed to harmonically oscillating light of low, medium, and high amplitudes, as described above. The sequence of oscillating light periods was similar to Niu et al. (2023), consisting of eight different periods that varied continuously from 8 min to 1 s: three oscillation cycles with an 8 min period, five cycles each with 4 min, 2 min, 1 min, 30 s, and 10 s periods, and finally ten cycles with 5 and 1 s periods. The light was controlled by an 8‐bit digital‐to‐analog converter, yielding 256 light levels to cover the amplitude range of the light intensities. This led to light changes in which discrete steps approximated the sine function: the oscillations were approximated by eight light intensity steps for 100–200 μmol photons m^−2^ s^−1^, 22 light intensity steps for 100–400 μmol photons m^−2^ s^−1^, and 49 light intensity steps for 100–800 μmol photons m^−2^ s^−1^. The light oscillation protocol and chlorophyll fluorescence responses are illustrated in Figure 1. Three plants of each *A. thaliana* genotype were measured in three oscillating light conditions as biological replicates.

The dynamic patterns of ChlF(*t*) signals sometimes change during the first one or two cycles following the change of the oscillation period. Therefore, only the steady‐state dynamic patterns^3^ that emerged in later cycles were used for the analysis. Specifically, the first cycle of the *T* = 8 min oscillation and the first two cycles of the other oscillation periods were excluded from the study to minimize aperiodic transient components.

The steady‐state ChlF(*t*) dynamic patterns were then averaged and fitted by the function in Equation (1) as previously done (Nedbal and Lazár 2021; Niu et al. 2023).

(1) $$\begin{array}{c} \mathrm{Fit}\left(t\right)=A_{0}+A_{1}\cdot \sin\left[1\cdot \frac{2\pi \left(t-\tau_{1}\right)}{T}\right]+A_{2}\cdot \sin\left[2\cdot \frac{2\pi \left(t-\tau_{2}\right)}{T}\right]\\ \;+A_{3}\cdot \sin\left[3\cdot \frac{2\pi \left(t-\tau_{3}\right)}{T}\right]+A_{4}\cdot \sin\left[4\cdot \frac{2\pi \left(t-\tau_{4}\right)}{T}\right] \end{array}$$

The least‐square fitting was done by Microsoft Excel, yielding the offset *A*_0_ as well as the amplitudes (*A*_1_, *A*_2_, *A*_3_, *A*_4_) and the phase shifts (*τ*_1_/*T*, *τ*_2_/*T*, *τ*_3_/*T*, *τ*_4_/*T*) of up to the fourth harmonic component. These nine parameters characterizing each of the three biological replicates separately were averaged, and statistical errors were calculated.

Figures SI‐1 to SI‐5 show that fitting by the analytical function in Equation (1) did not distort the ChlF loops.

### 2.2 Mathematical Model BDM2

**FIGURE 2:**
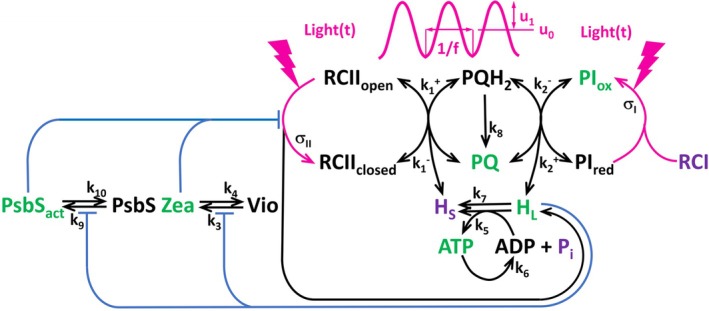
A scheme of the new version of the DREAM model (BDM2) that, in contrast to the parent version BDM (Fuente et al. 2024) differentiates between PsbS‐ and VDE‐dependent mechanisms of qE. The magenta‐colored sinusoidal curve represents harmonic light modulation. The oscillations of amplitude u_1_ and period *T* = 1/*f* (*f* is the frequency) are superimposed on the constant light level *u*_0_. The green color marks the independent variables. The black color is used for dependent variables and model parameters. The purple color marks the model variables assumed to be constant in time.

The model BDM2 used here for the in silico simulations is an upgrade of the parent BDM model (Fuente et al. 2024). The BDM2 scheme is shown in Figure 2. The most significant modification is the inclusion of the two mechanisms of NPQ regulation in BDM2 instead of the single mechanism considered in BDM. Two separate NPQ mechanisms in BDM2 allowed for representing the different kinetics of the PsbS‐ and zeaxanthin‐related NPQ mechanisms. The added dimension allowed for better simulations of ChlF dynamics with BDM2 than those that one could obtain with BDM.

BDM2 consists of six ordinary differential equations representing the redox state of the PQ pool by the PQ(*t*) variable, the redox state of the photosystem I donors by the PI_ox_(*t*) variable, the lumen proton concentration by the H_L_(*t*) variable, the ATP concentration by the ATP(*t*) variable, the zeaxanthin by the Zea(*t*) variable, and the protonated PsbS by the PsbS_act_(*t*) variable, all functions of time during light oscillation. The two new independent variables Zea(*t*) and PsbS_act_(*t*) replace in BDM2 the variable FQact(*t*) of the parent BDM model (Fuente et al. 2024). The activation dynamics of Zea(*t*) and PsbS_act_(*t*) are modeled similarly to eq. (11) and (12) in Fuente et al. (2024) by

(2) $$v_{3}\left(t\right)=k_{3}\cdot \left[1-\mathrm{Zea}\left(t\right)\right]\cdot \frac{1}{1+{\left[\frac{K_{Q,\mathrm{VDE}}}{H_{L}\left(t\right)}\right]}^{n_{\mathrm{VDE}}}}$$

and

(3) $$v_{9}\left(t\right)=k_{9}\cdot \left[1-{\text{PsbS}}_{\mathrm{act}}\left(t\right)\right]\cdot \frac{1}{1+{\left[\frac{K_{Q,\text{PsbS}}}{H_{L}\left(t\right)}\right]}^{n_{\text{PsbS}}}}$$

In addition to the two independent quenching mechanisms, we also modified some model parameters in BDM2. The complete list of model parameters used here in the simulations is in Table SI‐1. Most significantly, the values of the lumped rate constants *k*_1_^+^ and *k*_1_^−^ that approximately represent the forward and backward steady‐state electron transfer between PSII and the PQ pool in (Fuente et al. 2024) were too high, which led to predictions of unrealistically high rates of electron transport in saturating light. In BDM2, the values used for the simulations here are: *k*_1_^+^ = 250 s^−1^ and *k*_1_^−^ = 100 s^−1^. With these parameters, BDM2 predicts the rate of the steady‐state O_2_ evolution by the water splitting in PSII in saturating light (1000 μmol photons m^−2^ s^−1^) to be ≈ 60 O_2_ PSII^−1^ s^−1^, which is within the range of 27–67 O_2_ PSII^−1^ s^−1^ reported in the literature for saturating light (reviewed in Vinyard et al. 2013).

The rate of ATP formation, v_5_(*t*), described in Fuente et al. (2024) by Equation (16), was in BDM2 defined as

(4) $$v_{5}\left(t\right)=k_{5}\cdot \left\{\left[A_{\mathrm{tot}}-\mathrm{ATP}\left(t\right)\right]-a\cdot \frac{\mathrm{ATP}\left(t\right)}{{\left[H_{L}\left(t\right)\right]}^{\frac{14}{3}}}\right\}$$

where *a* = 9.202 × 10^−2^ is an empirical proportionality constant, with which the rate of steady‐state ATP production in saturating light (1000 μmol photons m^−2^ s^−1^) is ≈ 93 ATP ATP‐synthase^−1^ s^−1^. Considering the ATP‐synthase/PSII stoichiometry of 1/2 (Antal et al. 2013) and the fact that BDM2 includes only the linear electron transport, the rate is close to the maximal rate of 375 ATP ATP‐synthase^−1^ s^−1^ reported in the literature (Kocks and Ross 1995).

Further, the effective rate constants approximating violaxanthin deepoxidation to zeaxanthin and the reverse process were set to *k*_3_ = 0.01 s^−1^ and *k*_4_ = 0.001 s^−1^. The rate constants characterizing the activation and deactivation of the PsbS were set to *k*_9_ = 0.05 s^−1^ and *k*_10_ = 0.004 s^−1^. The pK values of the quencher activation were $K_{Q,\mathrm{VDE}}$ and $K_{Q,\text{PsbS}}$ were both set to 1 μM. The Hill dependence of the quencher activation was assumed to be slightly steeper for zeaxanthin *n*_VDE_ = 6 and slightly flatter for PsbS *n*_PsbS_ = 4 than assumed in the earlier model version, where *n* = 5.3 was used. The used values of the rate constants, pKs, and Hill coefficients are within the range reported in the literature (Jahns et al. 2001; Zaks et al. 2012; Matuszyńska et al. 2016; Steen et al. 2020; Short et al. 2022). The maximal extent of quenching was assumed in the model calculations for the WT to be Zea_max_ = 0.3 and PsbS_max_ = 0.3, for the *npq1* mutant to be Zea_max_ = 0 and PsbS_max_ = 0.3, and for the *npq*4 mutant to be Zea_max_ = 0.3 and PsbS_max_ = 0. These values were used to get values of the NPQ parameter simulated for saturating light by BDM2 similar to the experimentally measured values. The rate constant of ATP consumption, *k*_6_, was set to 8 s^−1^. As shown below in the Results section, these model parameters led to a good qualitative agreement between the model‐simulated ChlF dynamics and those obtained in the experiments with the WT plants. It is important to note that the parameter values can be further modified to reach even better agreement between the experiment and the simulations but this would not change any of our conclusions here. We preferred conserving most of the parameters of the parent BDM for the sake of an easier comparison.

The inactive violaxanthin deepoxidation in *npq1* was simulated by reducing the rate constant k_3_ by a factor of 1000 relative to WT. Similarly, the inactive PsbS‐dependent quenching in *npq4* was simulated by decreasing the rate constant k_9_ by a factor of 1000 relative to WT.

To compare the BDM2 simulations with experiments, we normalized the instantaneous chlorophyll fluorescence signal measured by the PAM method, *F*′(*t*), to the maximum *F*_M_ attained in a multiple‐turnover saturating flash in a dark‐adapted plant. With these modifications, one obtains for the normalized instantaneous chlorophyll fluorescence yield ChlF(*t*) the following expression:

(5) $$\begin{array}{c} \text{ChlF}\left(t\right)≝\frac{F\prime \left(t\right)}{F_{M}}=\left(1-{\mathrm{Zea}}_{\max}\cdot \mathrm{Zea}\left(t\right)\right)\cdot \left(1-{\text{PsbS}}_{\max}\cdot {\text{PsbS}}_{\mathrm{act}}\left(t\right)\right)\\ \;\cdot \left[\frac{\;1-{\text{RCII}}_{\text{closed}}\left(t\right)}{1+\frac{\Phi_{\text{IImax}}}{1-\Phi_{\text{IImax}}}\cdot \left(1-{\mathrm{Zea}}_{\max}\cdot \mathrm{Zea}\left(t\right)\right)\cdot \left(1-{\text{PsbS}}_{\max}\cdot {\text{PsbS}}_{\mathrm{act}}\left(t\right)\right)}+{\text{RCII}}_{\text{closed}}\left(t\right)\right] \end{array}$$

where RCII_closed_(*t*) is the dependent variable in BDM2 that represents the fraction of the closed reaction centers of PSII, and Zea(*t*) and PsbS_act_(*t*) are two of the six independent variables that represent fractions of the respective quenchers. Zea_max_ and PsbS_max_ are model parameters corresponding to the maximal NPQ when the respective quenchers are fully active. Ф_IImax_ (≝ $\frac{F_{V}}{F_{M}}$) is the maximum quantum yield of PSII photochemistry determined in the dark‐adapted state, where *F*_V_ = *F*_M_ − *F*_0_ is the maximal variable chlorophyll fluorescence yield in the dark‐adapted state (reviewed in Lazár 2015). The details of the Equation (5) derivation, using the approximation for *F*_0_′(*t*) (Oxborough and Baker 1997) are provided in the Supporting Information.

## 3 Results

### 3.1 The ChlF(*t*) Dynamics in the WT *A. thaliana* Col‐0

**FIGURE 3:**
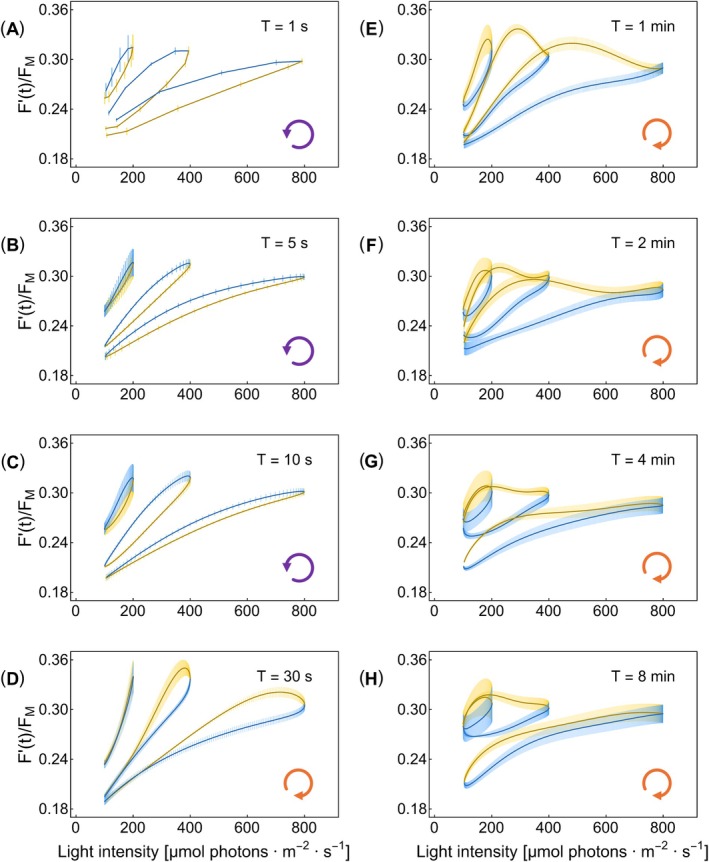
The ChlF(*t*) = *F*′(*t*)/*F*_M_ dynamics in the WT *A. thaliana* Col‐0. The steady‐state dynamics of ChlF(*t*) is shown as a function of light intensity, PAR. The light oscillated with eight different periods and three amplitudes (three different yellow–blue loops in each of the eight panels). The dynamics represent the means of three independent biological replicates, with error bars indicating standard errors (*n* = 3). The oscillation periods are noted in the legend of each panel, while the oscillating light intensity range for each loop is seen in the abscissa axis. The yellow–blue color code is the same as in Figure 1, and the loop arrows at the bottom right corner of each panel indicate the orientation of the loops.

The experimental results obtained with the WT *A. thaliana* Col‐0 are shown in Figure 3. The plots were obtained by calculating *A*_0_, *A*_1_, *A*_2_, *A*_3_, *A*_4_, and *τ*_1_/*T*, *τ*_2_/*T*, *τ*_3_/*T*, *τ*_4_/*T* in Equation (1) for each experimental dataset, followed by averaging and computing standard errors based on measurements on three plants. The average values of the parameters were used in Equation (1) to find the analytical representation of the data plotted by the solid lines in Figure 3. The error bars in Figure 3 show the variability of the analytical functions that originated from the experimental errors. The details of raw data, averages, and standard errors are shown in Figure SI‐1. The individual panels in Figure 3 represent the normalized steady‐state dynamic ChlF(*t*) response pattern of the plants exposed to actinic light oscillating with periods *T* = 1 s (A), 5 s (B), 10 s (C), 30 s (D), 1 min (E), 2 min (F), 4 min (G), and 8 min (H). Each panel shows three curves corresponding to light oscillating between 100 and 200, 100 and 400, and 100 and 800 μmol photons m^−2^ s^−1^ of PAR. As in Figure 2, each plotted curve consists of two phases: the first one when the light intensity oscillation starts at 100 μmol photons m^−2^ s^−1^ and gradually increases (ascending phase, yellow line) up to the maximum value (200, 400, or 800 μmol photons m^−2^ s^−1^) and the second one when the light intensity decreases back to 100 μmol photons m^−2^ s^−1^ (descending phase, blue line). The orientation of the loop is further emphasized by the purple, counter‐clockwise, and orange, clockwise arrows in Figure 3. The results demonstrate that ChlF(*t*) responses strongly depend on the oscillating light's period and amplitude.

The signal loops in Figure 3 mostly show two ChlF(*t*) values for the same incident light intensity, depending on the illumination history; such behavior is called hysteresis. The term hysteresis is derived from the ancient Greek expression for “lagging behind”. The hysteresis loops in Figure 3 differ depending on whether the delay arises from constitutive photosynthetic processes or the regulatory responses. These two types of hysteresis will be categorized as constitutive and regulatory, respectively.

The ChlF(*t*) response lagged the oscillating light when the periods were shorter than 30 s, that is, ChlF(*t*) was lower in the ascending light oscillation phase than in the descending phase for the same light intensity (Figure 3A–C). Such a delayed response is observed in many biological, chemical, and physical systems (Mayergoyz 2003; Strogatz 2018). We shall show further that this type of hysteresis occurs in all investigated plants, regardless of whether their NPQ mechanisms are fully intact, as in the WT, or compromised, as in the mutants. Such constitutive, regulation‐independent hysteresis occurs due to the delays in the primary photosynthetic reactions relative to rapidly changing light. The constitutive hysteresis of photosynthesis is rate‐dependent and, as such, decreases when the light oscillations are slower than the characteristic times of photosynthetic reactions. The loops representing the constitutive hysteresis in Figure 3A–C were nearly elliptical for the low‐ and medium‐amplitude oscillations (100–200 and 100–400 μmol photons m^−2^ s^−1^) and exhibited signs of saturation in the high‐amplitude oscillations (100–800 μmol photons m^−2^ s^−1^, Nedbal and Lazár 2021). The orientation of the constitutive hysteresis loops in Figure 3 is counter‐clockwise.

The ChlF(*t*) dynamics changed strikingly when the oscillation periods increased from *T* = 10 to 30 s (Figure 3D). The loop directions changed from counter‐clockwise, which were observed with shorter periods (Figure 3A–C), to clockwise orientation in the medium‐ and high‐amplitude oscillations of the 30‐s period (Figure 3D). This dynamic feature was observed in all light oscillation amplitudes, also with periods longer than 30 s (Figure 3E–H). In this case, ChlF(*t*) was higher in the light ascending phase than in the descending phase, and ChlF(*t*) started to decrease already in the light ascending phase, that is, the ChlF(*t*) maxima were reached before the light intensity peaked. This results from a delay in the regulatory response of NPQ, that is, from regulatory hysteresis. In our experiments with the high‐amplitude light oscillations, the regulatory hysteresis dominated in the period *T* = 1 min and decreased as the period further increased (Figure 3G,H). Thus, the ChlF(*t*) dynamics under high‐amplitude and slow oscillations converged to the typical steady‐state P–I curves, in which hysteresis is negligible because the photosynthesis apparatus has enough time to settle to the dynamic homeostasis for each light level, and little effect of the light history is therefore expected. Significant hysteresis remained even in the long periods (Figure 3G,H) when the low and medium oscillation amplitudes were applied. This may indicate that the mechanisms reducing hysteresis in the high‐amplitude oscillations and, therefore, the convergence to the steady‐state P–I, require high light. The shape of the dynamic pattern in the high‐light oscillations also supports this hypothesis.

### 3.2 The ChlF(*t*) Dynamics in the *A. thaliana npq1* and *npq4* Mutants

**FIGURE 4:**
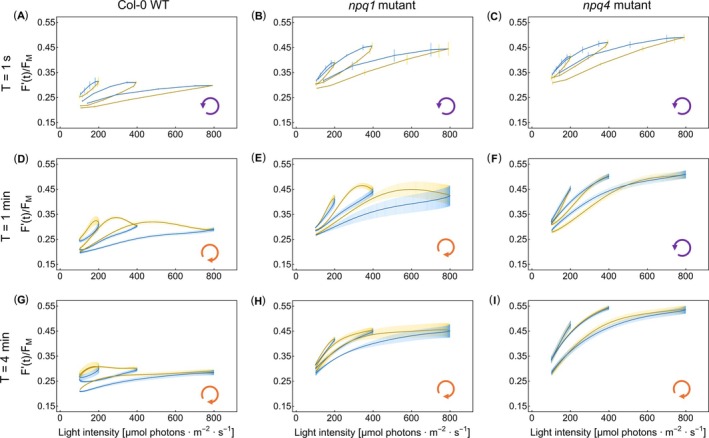
The dynamics of ChlF(*t*) = *F*′(*t*)/*F*_M_ in the *A. thaliana* Col‐0 WT (left panels A, D, G), *npq1* mutant (central panels B, E, H), and *npq4* mutant (right panels C, F, I). The top row panels (A–C) represent ChlF(*t*) dynamic patterns obtained with light oscillating with period *T* = 1 s, the middle row (D–F) with *T* = 1 min, and the bottom row (G–I) with *T* = 4 min. The yellow–blue color code is the same as in Figure 1. The loop arrows at the bottom right corner of each panel indicate the orientations of the loops.

The *npq4* mutant, which does not have the PsbS protein (Li et al. 2000), and the *npq1* mutant, which cannot convert violaxanthin into zeaxanthin (Niyogi et al. 1998), both exhibit ChlF(*t*) dynamic responses that differ from those of the Col‐0 WT (Figure 4). The top row of stimulus–response plots in Figure 4A–C shows the differences for the period *T* = 1 s that was associated with constitutive hysteresis in Figure 3. The dynamic patterns in the two lower rows (panels D–I), with *T* = 1 and 4 min, were dominantly formed by the regulatory hysteresis. The raw data and further details are shown in Figures SI‐3 and SI‐5.

The light oscillations with the short period of *T* = 1 s (Figure 4A–C) elicited ChlF(*t*) responses that were qualitatively similar in all three genotypes except for the average ChlF(*t*) levels, which were lower in the WT compared to the mutants. The WT plants were responding by NPQ that was partially incapacitated in the mutants, and therefore, the average ChlF(*t*) yield in the mutants was higher than that in the WT. As NPQ responded to the average light levels, the ChlF(*t*) in WT plants was the lowest in high‐amplitude oscillations. The opposite was the case for low‐amplitude oscillations. Except for this difference in the average ChlF(*t*) levels, the steady‐state dynamic patterns found in rapidly oscillating light in the WT and mutant plants were similar. This suggests that the *T* = 1 s loop is formed by constitutive rather than regulatory hysteresis.

In contrast to *T* = 1 s, it was the regulation that essentially formed the hysteresis found in the oscillations *T* = 1 and 4 min in the WT (Figure 4D,G) and in the *npq1* mutant (Figure 4E,H), a feature that was largely absent in the *npq4* mutant (Figure 4F,I). The experiment shows that regulatory hysteresis occurs due to the PsbS‐dependent qE that is active in the WT and *npq1*, but not in *npq4* plants. Regulatory hysteresis was weaker in *T* = 4 min than in *T* = 1 min, presumably because ChlF(*t*) was already approaching steady‐state in the long oscillation period, and, thus, the relative effects of regulations were less apparent with *T* = 4 min than with *T* = 1 min. The absence of zeaxanthin‐dependent qE in the *npq1* mutant and PsbS‐dependent qE in the *npq4* mutant led to higher amplitudes of the ChlF(*t*) patterns in Figure 4E,H, and Figure 4F,I compared to those in Figure 4D,G that represent the WT. This shows that both types of qE are required for dynamic homeostasis, which decides the stable levels of NPQ.

Overall, ChlF(*t*) in WT plants was much less sensitive to light oscillations than in the mutants: The stimulus–response patterns of the WT were nearly flat even when the oscillations reached high light levels (Figure 4G). This indicates that in the WT plants, the efficient pH‐dependent qE quenching balanced the light fluctuations and responded to the mean irradiance, enabling the system to maintain energetic homeostasis despite large changes in light intensity.

### 3.3 Comparing the ChlF(*t*) Dynamics Observed in Experiments With the BDM2 Model Simulations

**FIGURE 5:**
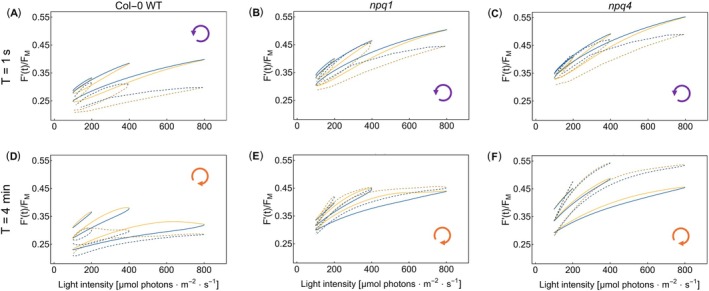
The experimentally measured ChlF(*t*) dynamics (dashed lines) presented in Figure 4 of WT *A. thaliana* Col‐0 (panels A and D) and of the *npq1* (panels B and E) and *npq4* (panels C and F) mutants are compared with simulations (solid lines) obtained with the BDM2, in which both the Zea(*t*) and PsbS_act_(*t*) quenchers were included (panels A and D) or the Zea(*t*) (panels B and E) or PsbS_act_(*t*) (panels C and F) quenchers were set to zero. The data and simulations were obtained with the short and long light oscillation periods, which have been shown to lead to the constitutive (*T* = 1 s) and regulatory (*T* = 4 min) hysteresis. The color code and symbols are the same as in the previous figures.

The experimentally measured ChlF(*t*) responses are compared with those simulated by BDM2 in Figure 5. Dashed lines represent the experimental data, and the simulations are shown by full lines. The comparison is done for short, *T* = 1 s, and long, *T* = 4 min periods of the light oscillations that lead to constitutive and regulatory types of hysteresis, respectively.

The ChlF dynamics of WT in the rapidly oscillating light (*T* = 1 s) are delayed relative to the forcing light phase. This delay originates in the primary photosynthetic processes. It is, therefore, called constitutive hysteresis with a counter‐clockwise orientation of the stimulus–response loops (Figure 5A). The same was also observed in the *npq1* (Figure 5B) and *npq4* (Figure 5C) mutants, confirming that constitutive hysteresis occurs irrespective of the qE mutations, irrespective of regulation. The constitutive hysteresis was found in the experiments (dashed lines in Figure 5) and confirmed by the BDM2 simulations (solid lines in Figure 5). The model simulations were obtained by including both the Zea(*t*) and PsbS_act_(*t*) model variables for WT (Figure 5A), or by setting very small values of the rate constants of formation of Zea in the *npq*1 mutant or of PsbS_act_ in the *npq*4 mutant, leading practically to no formation of Zea(*t*) (Figure 5B) or PsbS_act_(*t*) (Figure 5C) for the *npq*1 or *npq*4 mutants, respectively.

The ChlF(*t*) dynamics of WT in the slowly oscillating light (*T* = 4 min) were formed primarily by the delay of the qE regulation response relative to the forcing light phase. The delayed response of the qE regulation resulted in ChlF(*t*) pattern that was influenced by increasing quenching only later in the ascending phase of the light (yellow color line segments). Analogous regulatory delays formed the ChlF(*t*) response during the descending light phase (blue color line segments). The regulatory hysteresis was strongly expressed in the WT data as well as simulations (Figure 5D). It led to the clockwise orientation of the stimulus–response loops.

Regulatory hysteresis was also observed and simulated with the *npq1* mutant that was competent in the PsbS‐dependent quenching but lacked the VDE mechanism (Figure 5E). The regulatory hysteresis can, therefore, be linked to the PsbS‐dependent quenching. The absence of VDE‐dependent regulation was expressed by the ChlF(*t*) levels that were significantly higher in the *npq1* mutant (Figure 5E) than in WT (Figure 5D). This suggests that the VDE‐dependent quenching acts in the WT on average ChlF yield over periods much longer than 4 min.

Regulatory hysteresis was barely seen in the ChlF(*t*) responses to the slowly oscillating light in the *npq4* mutant that lacks the PsbS quenching capacity (Figure 5F). Also, the average ChlF(*t*) levels were higher in the *npq4* mutant than in the *npq1* mutant and in the WT plants. This suggests that regulatory protection against oscillating light is largely incapacitated in the *npq4* mutant.

### 3.4 Molecular Mechanisms Shaping the ChlF(*t*) Responses to Oscillating Light

**FIGURE 6:**
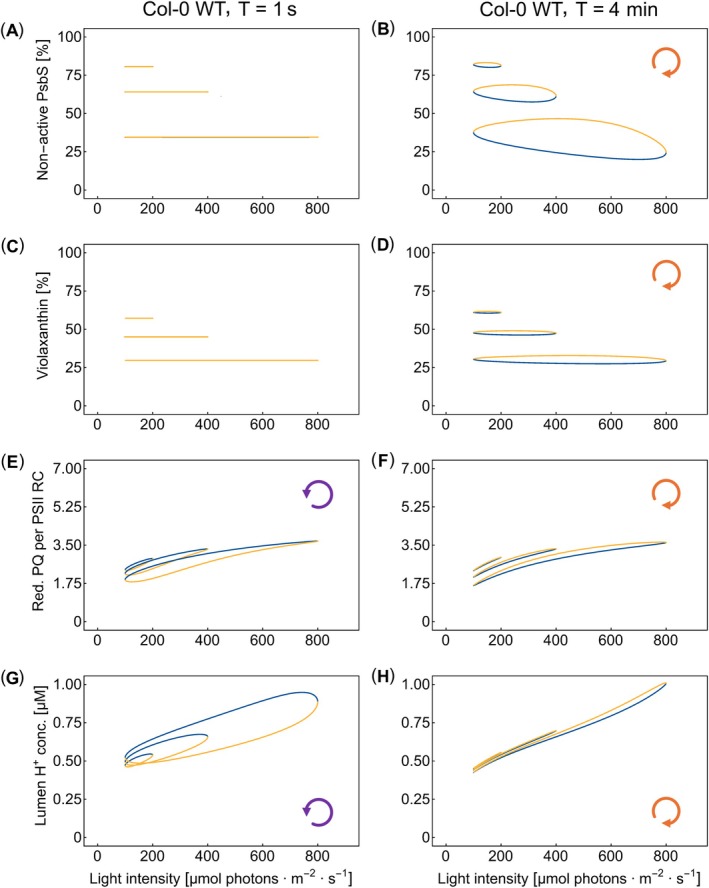
The simulated dynamics of non‐active PsbS (=1 − PsbS_act_(*t*), panels A, B), of violaxanthin (=1 − Zea(*t*), panels C, D), of reduced PQ pool per PSII reaction center (=PQ_tot_ − PQ(*t*), panels E, F), and of the lumen H^+^ concentration (panels G, H) of the WT. The panels of the left column show simulations for oscillation periods *T* = 1 s, while those of the right column panels depict simulations for oscillation periods *T* = 4 min. The color code and symbols are the same as in the previous figures.

The dynamics of the inactive forms of the quenchers, violaxanthin (1 − Zea(*t*)) and the inactive form of PsbS (1 − PsbS_act_(*t*)), and reduced PQ (PQ_tot_ − PQ(*t*)), which are expected to be largely positively correlated with ChlF(*t*), were simulated and are shown together with the concentration of H^+^ in the lumen in Figure 6. The dynamics in WT are distinctly different in the fast and slow light oscillations. The differences can be used to understand the molecular mechanisms forming the constitutive (*T* = 1 s) and regulatory (*T* = 4 min) hysteresis types. The analogous simulations for the *npq1* and *npq4* mutants are compared with the WT simulations in Figure SI‐6.

The simulations confirm that the PsbS‐ and VDE‐responses were too slow to follow the rapid light oscillations (*T* = 1 s). The non‐active PsbS (Figure 6A) and violaxanthin (Figure 6C) remained constant during the short oscillation period in all three organisms (Figure SI‐6). The quenchers could not activate and deactivate within 1 s, although the lumenal H^+^ oscillated strongly (Figure 6G). This explains the absence of regulatory hysteresis in ChlF(*t*) with *T* = 1 s in the WT (Figure 5A). The same conclusions are also valid for non‐active PsbS in the *npq1* mutant (Figure SI‐6B) and violaxanthin in the *npq4* mutant (Figure SI‐6I), and the absence of the regulatory hysteresis in ChlF(*t*) for *T* = 1 s in the mutants (Figure 5B,C).

In contrast, the non‐active PsbS and violaxanthin exhibited in WT a strong hysteresis with higher values in the ascending than in the descending phase of the light oscillation when the oscillations were slow, *T* = 4 min (Figure 6B,D). The maxima of the non‐active PsbS and violaxanthin occur deep in the ascending light phase, which means that ChlF(*t*) maximum occurs before the light maximum. This is because the regulatory response is delayed after the light oscillation. The same is also true for the mutants (Figure SI‐6E,L).

Interestingly, the PQ pool of the WT and *T* = 4 min is also more reduced in the ascending than in the descending light phase (Figure 6F). Thus, the regulatory hysteresis of the quenching is translated with *T* = 4 min into the constitutive process of PQ reduction, overriding the constitutive hysteresis of the PQ reduction that was found with *T* = 1 s. ChlF(*t*) of WT thus shows with *T* = 4 min the regulatory‐type hysteresis (Figure 5D) with the clockwise orientation of the hysteresis loop.

The lumen H^+^ concentration response of the WT in long‐period oscillations, *T* = 4 min, was nearly synchronized with light intensity changes (Figure 6H). Since the more PQ pool is reduced, more protons are released to the lumen. The orientation and width of H^+^ concentration hysteresis loops (Figure 6G,H) followed the behavior of the reduced PQ pool hysteresis loops (Figure 6E,F) for both the long and short periods of the oscillating light.

## 4 Discussion

### 4.1 The Constitutive Hysteresis of ChlF(*t*) Reflects a Kinetic Limitation in the Electron Transport Chain

The dynamics of ChlF(*t*) in the rapidly oscillating light (*T* ≤ 10 s) exhibit constitutive hysteresis, characterized by a delayed ChlF(*t*) response relative to the light stimulation occurring in WT plants (Figure 3A–C) as well as in the mutants (Figure 4A–C). It is the filling and emptying of the primary reactant pools (Figure 6E, Figure SI‐6M–O; Nedbal and Koblížek 2006; Rascher and Nedbal 2006; Kalaji et al. 2012), not the regulation (Figure 6A,C; Figure SI‐6A–C,G–I) that causes the delayed response and the constitutive hysteresis.

As suggested in Nedbal and Lazár (2021), this dynamic behavior is homologous to that of a resistor‐capacitor electronic circuit in which the capacitor charging represents the accumulation of reduced PQ or acidification of the thylakoid lumen. The homology suggests that decreasing the light oscillation period below 1 s will decrease the amplitude of the ChlF(*t*) response, as with the low‐pass electronic filter (Nedbal and Lazár 2021). For much slower light oscillations, here *T* = 4 min, the PQ pool reduction and the thylakoid lumen acidification would, in the absence of regulation, follow the light modulation without any constitutive hysteresis.

The ChlF(*t*) response is in the high‐light range constrained by the limited size of the PQ pool and by the saturation of the photosynthetic reactions. This constitutive non‐linearity is another dynamic feature forming the ChlF(*t*) response in high‐light oscillations in WT and mutants.

It is important to note that, although the qE regulation cannot keep pace with the rapidly oscillating light, it responds to the average light intensity (Figure 6A,C). The activation of the quenchers is high in light that oscillates between 100 and 800 μmol photons m^−2^ s^−1^ and decreases with the oscillation maxima dropping to 400 and 200 μmol photons m^−2^ s^−1^ (Figure 6A,C; note that the panels show 1 − PsbS_act_(*t*), and 1 − Zea(*t*), respectively).

### 4.2 The Regulatory Hysteresis of ChlF(*t*) Reflects a Delay in the qE Response

The qE regulation in WT plants could follow the light oscillations when the periods were *T* = 30 s and longer. The quenching, however, lagged the light oscillation, which led to the ChlF(*t*) maximum occurring before the maximum of the light and, therefore, to a change of the ChlF(*t*) loop orientation. The ChlF(*t*) loop orientation changed from counter‐clockwise with the short oscillation periods (Figure 3A–C) to clockwise with the long periods (Figure 3D–H) because the latter response was primarily formed by the qE regulation (Figure 6B,D). This is in agreement with the measurements that applied saturated flashes during the light oscillations and that revealed a delay of the qE regulation of about 15 s during the ascending light phase of *T* = 1 min light oscillations (Shimakawa and Miyake 2018; Lazár et al. 2022; Niu et al. 2024).

The oscillation period of 1 min was already long enough for extensive periodic activation and deactivation of the quenching mechanisms and, yet, still comparable to the lag in the regulatory response (Shimakawa and Miyake 2018; Lazár et al. 2022; Niu et al. 2024). This made the regulatory hysteresis in the loop in Figure 3E dominant. Further increasing the oscillation period to several minutes in Figure 3F–H led, particularly in the high‐light range, to the narrowing of the hysteresis loops, which can be explained by a fully developed qE that can follow the slowly oscillating, strong light with a negligible delay.

The regulatory hysteresis dominated the ChlF(*t*) response to 1‐min light oscillations not only in the WT (Figure 4D) but also in the *npq1* mutant (Figure 4E), both competent in the PsbS‐dependent qE. The absence of a similarly strong hysteresis in the *npq4* mutant (Figure 4F) indicates that the observed regulatory hysteresis depends on the dynamics of PsbS protein activation and deactivation. The *npq1* mutant (Figure 4E) exhibited higher average ChlF(*t*) than the WT (Figure 4D), suggesting that zeaxanthin‐dependent qE, though not dominating the regulatory dynamic response, reduces the amplitude of ChlF(*t*) changes and suppresses oscillation in the photosynthesis system under oscillating light. However, zeaxanthin‐dependent qE alone fails to induce effective dynamic regulation in the absence of PsbS protein for the period *T* = 1 min (Figure 4F).

Constitutive and regulatory hysteresis were observed in the WT in high‐ as well as in low‐light oscillations (Figure 4A,D,G). Constitutive hysteresis was observed in the *npq1* and *npq4* mutants also with all light oscillation amplitudes when the oscillation periods were short (Figure 4B,C), confirming that the phenomenon depends on the primary reactions, not on qE regulation. Interestingly, however, the regulatory hysteresis that was apparent in the *npq1* mutant in the high‐ and medium‐amplitude light oscillations was almost absent when the light oscillated only in the sub‐saturating intensity range (Figure 4E and Figure SI‐5E–H). This may be tentatively interpreted by zeaxanthin's role in modulating the relationship between qE and lumen pH (Noctor et al. 1991, 1993). Zeaxanthin may act as an allosteric modulator of qE, altering its efficiency and kinetics by shifting the apparent pK of qE from 4.5 to 6.5 or a more alkaline pH (Crouchman et al. 2006; Johnson et al. 2008, 2009; Pérez‐Bueno et al. 2008; Johnson and Ruban 2009). Existing zeaxanthin in WT plants enables qE activation at a higher lumen pH, which typically occurs in low‐light oscillation, whereas in the *npq1* mutant, the absence of zeaxanthin necessitates a lower lumen pH to activate qE. These findings support the idea that zeaxanthin plays a regulatory role in qE response, and that the relationship between qE and ΔpH is non‐linear and dynamically altered (Noctor et al. 1991; Niyogi et al. 1998; Johnson et al. 2008; Nilkens et al. 2010; Jahns and Holzwarth 2012).

In a previous paper (Niu et al. 2023), we proposed that in high‐light oscillation with tested periods (100–800 μmol photons m^−2^ s^−1^; 1 s–8 min periods), zeaxanthin produced during the pre‐illumination and high‐light phases of oscillation cannot apparently decline during the relatively brief low‐light phases of oscillation. However, this may not apply to the low‐light oscillations studied here (100–200 μmol photons m^−2^ s^−1^). The re‐conversion of zeaxanthin to violaxanthin in darkness or low light depends on pre‐illumination intensity, with higher intensity slowing down the re‐conversion of zeaxanthin to violaxanthin by lowering the amount of zeaxanthin epoxidase through protein degradation (Jahns 1995; Jahns and Holzwarth 2012; Kress and Jahns 2017). Low‐light oscillations with long periods may allow the xanthophyll cycle to operate bidirectionally with ΔpH changes (Jahns 1995), leading to dynamic changes in zeaxanthin concentration. The potential oscillations in zeaxanthin concentration can directly affect qE, which could also explain the difference in ChlF(*t*) dynamics observed between the WT and *npq1* mutant in low‐light long‐period oscillations (Figure 4D,E,G,H). Further studies on the changes in xanthophyll composition and the proton motive force could clarify zeaxanthin's role in qE regulation under oscillating light.

The present findings suggest that biological regulation works well with a tandem of molecular mechanisms that include a rapid response, here by PsbS, with a more persistent regulator and regulation modulator, here by zeaxanthin.

## 5 The Conclusions and Outlook

The frequency domain approach to photosynthesis is still in its infancy relative to the vast range of applications in physics and engineering. It requires meticulous probing and validation of fundamental principles by comparing experimental data with predictions of mathematical models. The present study demonstrated that the improved mathematical model, BDM2, can accurately simulate experimental data obtained from WT plants, *npq1* and *npq4* mutants, and various light oscillation periods, encompassing transitions from linear proportionality to saturation. The *npq1* and *npq4* mutants, which are already extensively characterized in the time domain, served here as convenient reference organisms for developing and validating novel frequency domain approaches. The created tools will be further utilized to explore the synergies of more complex regulatory networks of photosynthesis operating in various plants and algae across different frequency domains.

The good qualitative agreement between the data and model simulations is significant because it was achieved despite the BDM2 model being highly simplified. Representing multiple complex processes, such as those in Photosystem II, with a single model variable, characterizing multiple processes with a single “lumped” rate constant limits the applicability of BDM2 across different timescales and for explaining a wider range of phenomena. Yet, the good qualitative agreement of the model simulations with experimentally found ChlF(*t*) dynamics reported here demonstrates the strength of the parsimony principle, keeping the model dimensionality at its minimum, which is sufficient to explain the phenomena observed in the experiments; the principle is also applied in Fuente et al. (2024). The model dimensionality reduction should be further exploited and extended for a detailed analysis of responses in a narrow frequency range, where, for example, only constitutive or only regulatory processes contribute to the dynamics of photosynthesis.

Intentionally, we have not performed any numerical parameter optimization aimed at further minimizing the differences between experimental data and model simulations. These differences are an essential guide for identifying potential structural deficiencies in our models. We expect that the differences will increase when the range of applied light frequencies is further extended and will enable substantial amendments to future models, considering linear electron transport beyond the PQ pool, photosynthesis control, cyclic electron transport, the Calvin‐Benson‐Bassam cycle, and other relevant processes. Yet, even then, the model dimensionality should be kept at the minimum necessary to explain phenomena occurring in the experiments within the selected frequency domain. Modeling oscillations occurring with periods of tens of minutes cannot be done without considering slow processes up to stomata responses or chloroplast movement, but at the same time, many fast processes can be ignored or lumped in such models.

The traditional empirical approach to mathematical modeling in photosynthesis research can also be supplemented in the future by advanced approaches, in which the differences between an existing model's predictions and experimental data are used to identify and characterize modules and regulations required to understand the plant response dynamics.

## Author Contributions

S.M. formulated the experimental plan and contributed to the final formulation of the manuscript. Y.N. performed the experiments, analyzed the experimental data, and described them in the initial version of the manuscript. D.F. participated in developing the model, did the computer simulations, described them in the initial version of the manuscript, and generated all figures. D.L. participated in developing the model and wrote the final manuscript version. L.N. formulated the approaches to the data analysis, designed the analysis routines, participated in developing the model, and wrote the final manuscript version.

## Supporting information


Data S1.

## Data Availability

Raw experimental data and BDM2 code are available upon request.

## Endnotes

^1^
It is sometimes also called the input–output relationship, as in Frank (2013).

^2^
Harmonic functions of various periods constitute a unique base in the Hilbert space of all functions that may represent various light fluctuating patterns (Schwartz 2008). They are unique by their orthogonality that might have an analogue in the orthogonality of the usual *X*‐*Y*‐*Z* Cartesian coordinates. The fact that the *X*‐, *Y*‐, and *Z*‐axis are perpendicular, that is, orthogonal means that every position in our 3D space can be characterized by 3 unique numbers (*x*, *y*, *z*). Similarly, any relevant function representing a light pattern can be represented by a linear combination of harmonic functions of various frequencies.

^3^
We use the term “steady‐state dynamic pattern” to describe the response of a system that exhibits continuous motion or oscillation while maintaining a constant overall behavior. Here, it was when *A*_0_, *A*_1_, *A*_2_, *A*_3_, *A*_4_, and *τ*_1_/*T*, *τ*_2_/*T*, *τ*_3_/*T*, *τ*_4_/*T* were not changing over time or when the changes were small and smoothed out by averaging over several oscillation periods.
