# Supplementary material for: Constitutive and Regulatory Responses of Arabidopsis thaliana to Harmonically Oscillating Light
Source: Physiol Plant. 2025 Jul 31;177(4):e70421. doi: 10.1111/ppl.70421 (PMC12312291; doi:10.1111/ppl.70421)
Supplement: Supplementary file 1 — Data S1. [file PPL-177-e70421-s001.pdf]

SUPPORTING INFORMATION

Constitutive and regulatory responses of *Arabidopsis thaliana* to harmonically oscillating light

Yuxi Niu, David Fuente, Shizue Matsubara, Dušan Lazár, Ladislav Nedbal

Parameters used in the BDM2 simulations of the ChlF(t) dynamics in WT plants

Table SI-1

Parameter	Value	Comment	Reference
nPSII	1	Stoichiometry of PSII	the same as in Fuente *et al.* (2024)
nPSI	1 / RCII	Stoichiometry of PSI	the same as in Fuente *et al.* (2024)
PQ_{tot}	7 / RCII	Stoichiometry of the PQ pool	the same as in Fuente *et al.* (2024)
$[H^+]_{stroma}$	$10^{-1.8}$ µM	Proton stromal concentration	the same as in Fuente *et al.* (2024)
A_{tot}	1000 µM	Adenylate stromal concentration (≈ ADP+ATP)	the same as in Fuente *et al.* (2024)
V_L	$2.62 \cdot 10^{-21}$ L / RCII	Lumen volume corresponding to one RCII	the same as in Fuente *et al.* (2024)
V_S	$2.09 \cdot 10^{-20}$ L / RCII	Stroma volume corresponding to one RCII	the same as in Fuente *et al.* (2024)
σ_{II}	1 s^{-1} (µmol photons m^{-2} s^{-1}) $^{-1}$	Effective antenna size of PSII	the same as in Fuente *et al.* (2024)
σ_{I}	1 s^{-1} (µmol photons m^{-2} s^{-1}) $^{-1}$	Effective antenna size of PSI	the same as in Fuente *et al.* (2024)
k_1^+	250 s^{-1}	Effectively representing all reactions involved in the electron transfer $RCII_{closed}(Q_A^-)$ → PQ	estimated, see the main text
k_1^-	100 s^{-1}	The rate-limiting step in all reactions involved in PQH_2 → $RCII_{open}$ (Q_A)	estimated, see the main text
k_2^+	100 s^{-1}	The rate-limiting step in all reactions involved in PQH_2 → PI_{ox}	the same as in Fuente *et al.* (2024)
k_2^-	10 s^{-1}	The rate-limiting step in all reactions involved in PI_{red} → PQ	the same as in Fuente *et al.* (2024)
k_3	0.01 s^{-1}	Formation of Zea by the de-epoxidation of violaxanthin	estimated, see the main text
k_4	0.001 s^{-1}	Zeaxanthin epoxidation	estimated, see the main text
k_5	100 s^{-1}	ATP formation	the same as in Fuente *et al.* (2024)
k_6	8 s^{-1}	ATP consumption	estimated, see the main text
k_7	500 s^{-1}	H^+ leakage and other mechanisms dissipating ΔH^+	the same as in Fuente *et al.* (2024)
k_8	1 s^{-1}	Reduction of O_2 by the plastid terminal oxidase	the same as in Fuente *et al.* (2024)
k_9	0.05 s^{-1}	Protonation of PsbS	estimated, see the main text
k_{10}	0.004 s^{-1}	De-protonation of PsbS	estimated, see the main text
$L_{1/2}$	10^4 µmol photons m^{-2} s^{-1}	Half-saturation of PSI	the same as in Fuente *et al.* (2024)
b_H	0.01	pH buffer in the lumen	the same as in Fuente *et al.* (2024)
Zea_{max}	0.30	Maximal extent of zeaxanthin-dependent NPQ	estimated, see the main text
n_{VDE}	6	Hill coefficient of zeaxanthin-dependent NPQ	estimated, see the main text
$K_{Q,VDE}$	1 µM	pK of the Zea accumulation	estimated, see the main text
$PsbS_{max}$	0.30	Maximal extent of PsbS-dependent NPQ	estimated, see the main text
n_{PsbS}	4	Hill coefficient of PsbS-dependent NPQ	estimated, see the main text
$K_{Q,PsbS}$	1 µM	pK of the PsbS protonation	estimated, see the main text
a	$9.202 \cdot 10^{-2}$	Proportionality constant	estimated, see the main text
N_A	$6.022 \cdot 10^{17}$ µmol^{-1}	Avogadro constant	-

The ChlF(t) formula (Eq. 5 in the main text)

Using the formula derived by Oxborough and Baker (1997), one can express this dependent variable normalized to F_M as:

$$\frac{F_0{}'(t)}{F_M} = \frac{\frac{F_0}{F_M}}{\frac{F_V}{F_M} + \frac{F_0}{F_M{}'(t)}} = \frac{\frac{F_M - F_V}{F_M}}{\frac{F_V}{F_M} + \frac{F_M - F_V}{F_M} \cdot \frac{F_M}{F_M{}'(t)}} = \frac{1}{\frac{\phi_{IImax}}{1-\phi_{IImax}} + \frac{F_M}{F_M{}'(t)}}, \qquad \text{Eq. SI-1}$$

where $\Phi_{IImax} = \frac{F_V}{F_M}$ is the maximum quantum yield for PSII photochemistry in the dark-adapted state and F_V and F_M are the variable and maximum fluorescence parameters measured by PAM techniques. Here, we use $\frac{F_V}{F_M} = 0.8$ (Lazár *et al.*, 1998).

The maximal chlorophyll fluorescence yield in the light-adapted state $F_M{}'(t)$ is smaller than in the dark-adapted state F_M. The reduction is assumed in the model to be due to the synergistic quenching by zeaxanthin and by activated PsbS:

$$F_M{}'(t) = F_M \cdot \left(1 - Zea_{max} \cdot Zea(t)\right) \cdot \left(1 - PsbS_{max} \cdot PsbS_{act}(t)\right) \qquad \text{Eq. SI-2}$$

The zeaxanthin formation is assumed in the model to result from violaxanthin de-epoxidation (VDE). The model ignores the formation of the antheraxanthin fraction. The model independent variable Zea(t) is 0 when no quencher is present and 1 when full de-epoxidation occurs. The zeaxanthin-dependent fluorescence quenching reaches its maximum by a factor of $(1 - Zea_{max})$ when Zea(t) = 1. Similarly, the independent variable $PsbS_{act}(t) = 0$ when the PsbS-dependent quenching is inactive and $PsbS_{act}(t) = 1$ for fully activated PsbS. The parameter values $Zea_{max} = 0.3$ and $PsbS_{max} = 0.3$ are model assumptions to get values of the NPQ parameter simulated for saturating light by BDM2 similar to the experimentally measured values. With this, the maximal quenching that the BDM2 model can simulate is $F_M{}'(t) = 0.49\ F_M$ (it was 0.4 F_M in Fuente *et al.*, 2024). By substituting Eq. SI-2 in Eq. SI-1, one obtains:

$$\frac{F_0{}'(t)}{F_M} = \frac{\left(1 - Zea_{max} \cdot Zea(t)\right) \cdot \left(1 - PsbS_{max} \cdot PsbS_{act}(t)\right)}{1 + \frac{\phi_{IImax}}{1-\phi_{IImax}} \cdot \left(1 - Zea_{max} \cdot Zea(t)\right) \cdot \left(1 - PsbS_{max} \cdot PsbS_{act}(t)\right)} \qquad \text{Eq. SI-3}$$

Similarly, the normalized relative maximal variable chlorophyll fluorescence yield for a light-adapted state can be expressed as:

$$\frac{F_V{}'(t)}{F_M} = \frac{F_M{}'(t) - F_0{}'(t)}{F_M} \cdot RCII_{closed}(t), \qquad \text{Eq. SI-4}$$

where $F_0'(t)/F_M$ and $F_M'(t)$ are given by Eqs. SI-1 and SI-2, respectively, and $RCII_{closed}(t)$ is the fraction of closed (with reduced Q_A) reaction centers of PSII at time t.

Adding $\frac{F_0'(t)}{F_M}$ from Eq. SI-3 and $\frac{F_V'(t)}{F_M}$ from Eq. SI-4, one obtains for total ChlF(t) Eq. 5 as it is used in the main text:

$$\mathrm{ChlF(t)} \stackrel{\text{def}}{=} \frac{F'(t)}{F_M} = \left(1 - \mathrm{Zea}_{max} \cdot \mathrm{Zea(t)}\right) \cdot \left(1 - \mathrm{PsbS}_{max} \cdot \mathrm{PsbS}_{act}(t)\right) \cdot \left[\frac{1 - \mathrm{RCII}_{closed}(t)}{1 + \frac{\Phi_{IImax}}{1 - \Phi_{IImax}} \cdot \left(1 - \mathrm{Zea}_{max} \cdot \mathrm{Zea(t)}\right) \cdot \left(1 - \mathrm{PsbS}_{max} \cdot \mathrm{PsbS}_{act}(t)\right)} + \mathrm{RCII}_{closed}(t)\right]$$

It is easy to verify that

- in the case of open reaction centers and no quenching:

$$\mathrm{ChlF(t)}^{open\ RC,\ no\ quenching} = \frac{F_0}{F_M} = 0.2$$

- in the case of closed reaction centers and no quenching:

$$\mathrm{ChlF(t)}^{closed\ RC,\ no\ quenching} = \frac{F_M}{F_M} = 1$$

- in the case of open reaction centers and the maximum quenching:

$$\mathrm{ChlF(t)}^{open\ RC,\ max\ quenching} = \frac{(1 - \mathrm{Zea}_{max}) \cdot (1 - \mathrm{PsbS}_{max})}{1 + \frac{\emptyset_{IImax}}{1 - \emptyset_{IImax}} \cdot (1 - \mathrm{Zea}_{max}) \cdot (1 - \mathrm{PsbS}_{max})} = \frac{F_0'(t)}{F_M} \approx 0.178$$

- in the case of closed reaction centers and the maximum quenching:

$$\mathrm{ChlF(t)}^{closed\ RC,\ max\ quenching} = \frac{F_M'(t)}{F_M} = 0.49$$

Raw ChlF experimental data on the Col-0 wildtype

Fig. 3 in the main text interpolates the ChlF dynamics by functions from Eq. 1 in which the amplitude and phase parameters were obtained by numerical fittings of individual experimental data sets. Fig. SI-1 represents the same data by averages and standard errors of three experiments. Comparing this figure with Fig. 3 confirms that the numerical fitting by the function Eq. 1 does not distort the ChlF(t) dynamic pattern observed in the experiments. The fitting is done to compare analytical functions characterizing the experiment and the simulations.

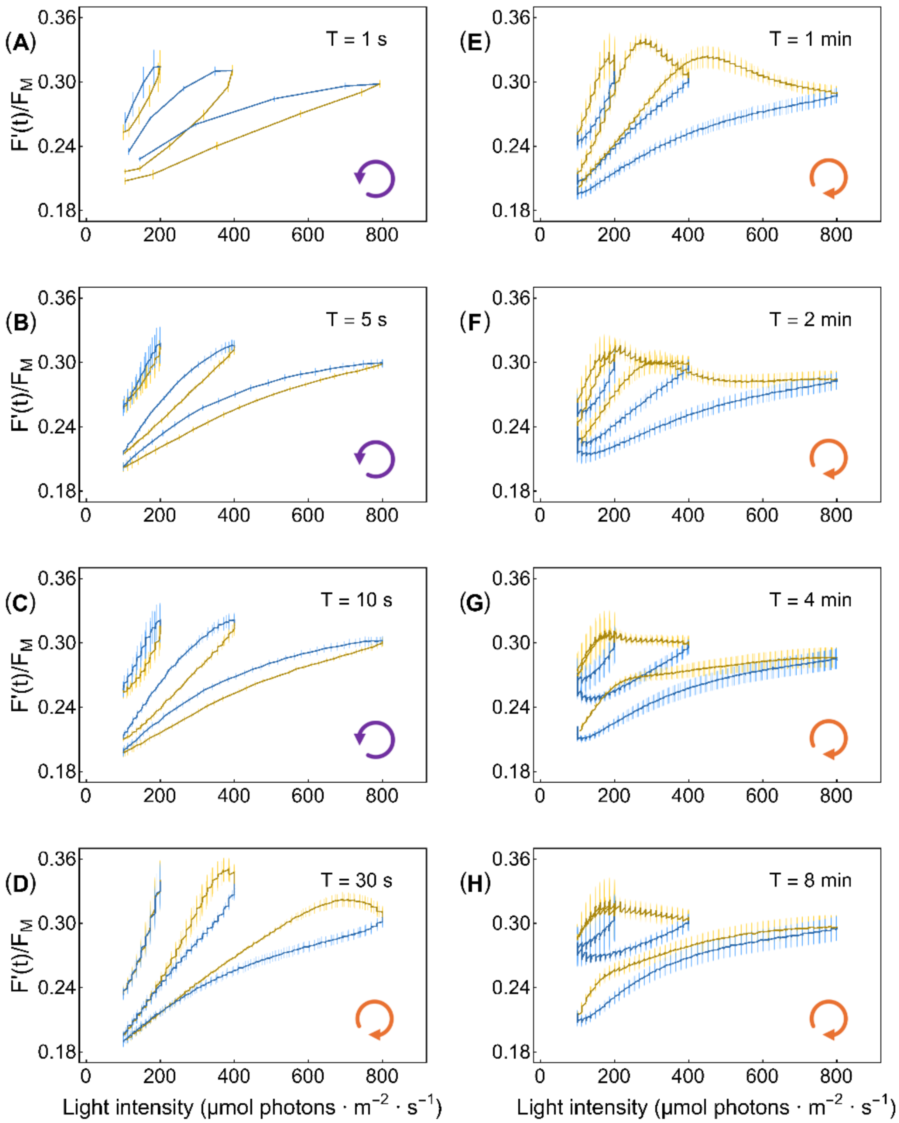

Figure SI-1 The dynamics of the original experimental data on ChlF(t) obtained with the WT *A. thaliana* Col-0. ChlF(t) is shown as a function of the intensity of PAR that oscillates with different periods and amplitudes. The dynamics represent an average of three independent biological replicates, with error bars indicating standard errors (n = 3).

Raw ChlF experimental data on the *npq4* mutant

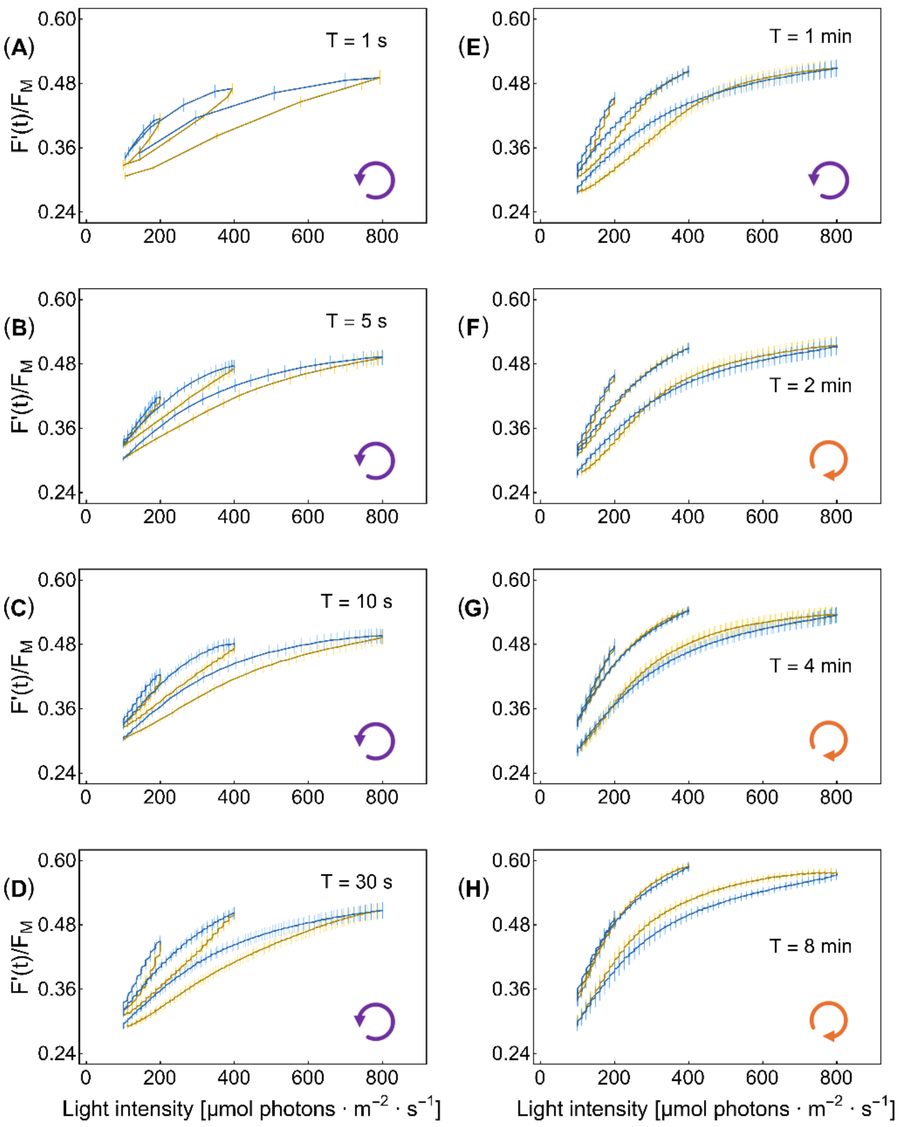

Figure SI-2 The dynamics of the original experimental data on ChlF(t) obtained with the *A. thaliana npq4* mutant. The marking and legends are the same as in Figure SI-1.

The ChlF experimental data on the *npq4* mutant fitted by the four harmonic components of the function in Eq. 1.

The agreement between Fig. SI-2 and SI-3 shows that the numerical fitting did not change the ChlF dynamic pattern.

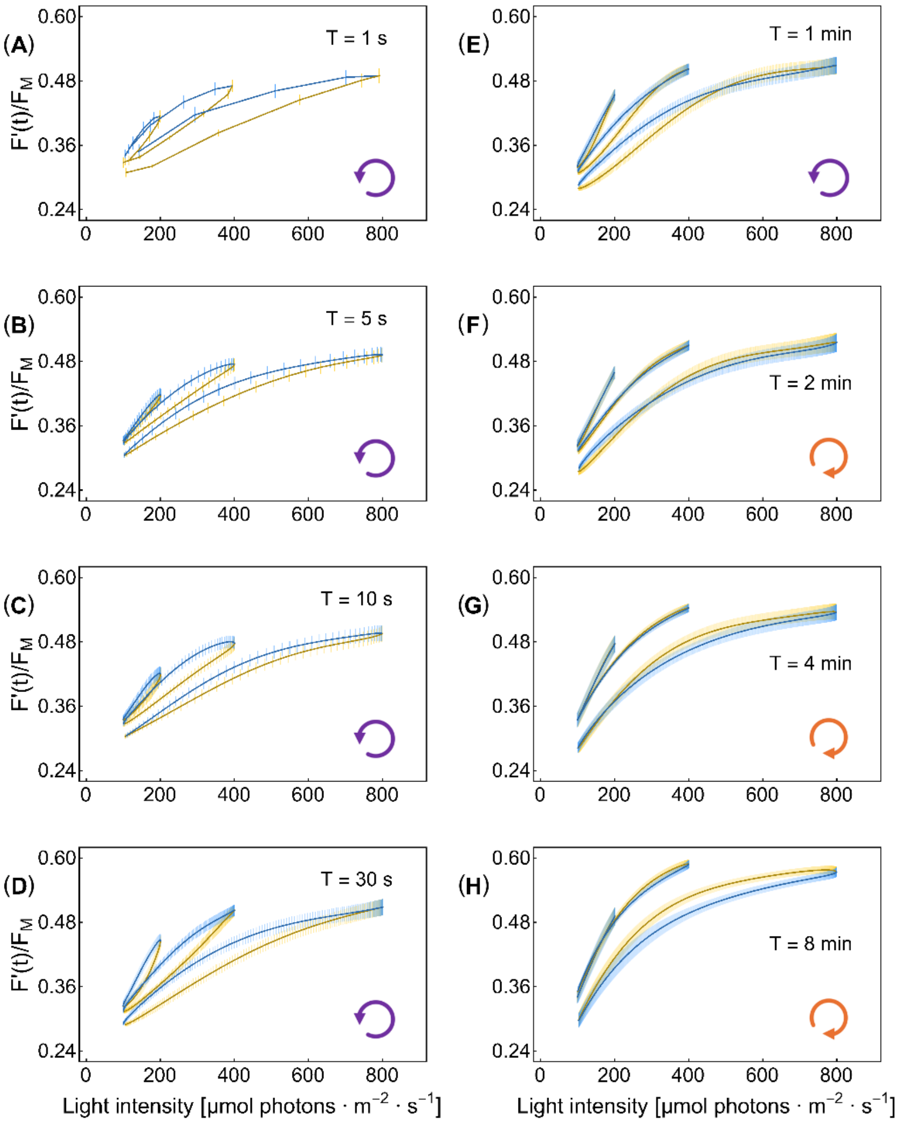

Figure SI-3 Data on *A. thaliana npq4* mutant represented in Figure SI-2 fitted by the function in Eq. 1, the fits averaged and analyzed for experimental error and represented here by resulting analytical function.

Raw ChlF experimental data on the *npq1* mutant

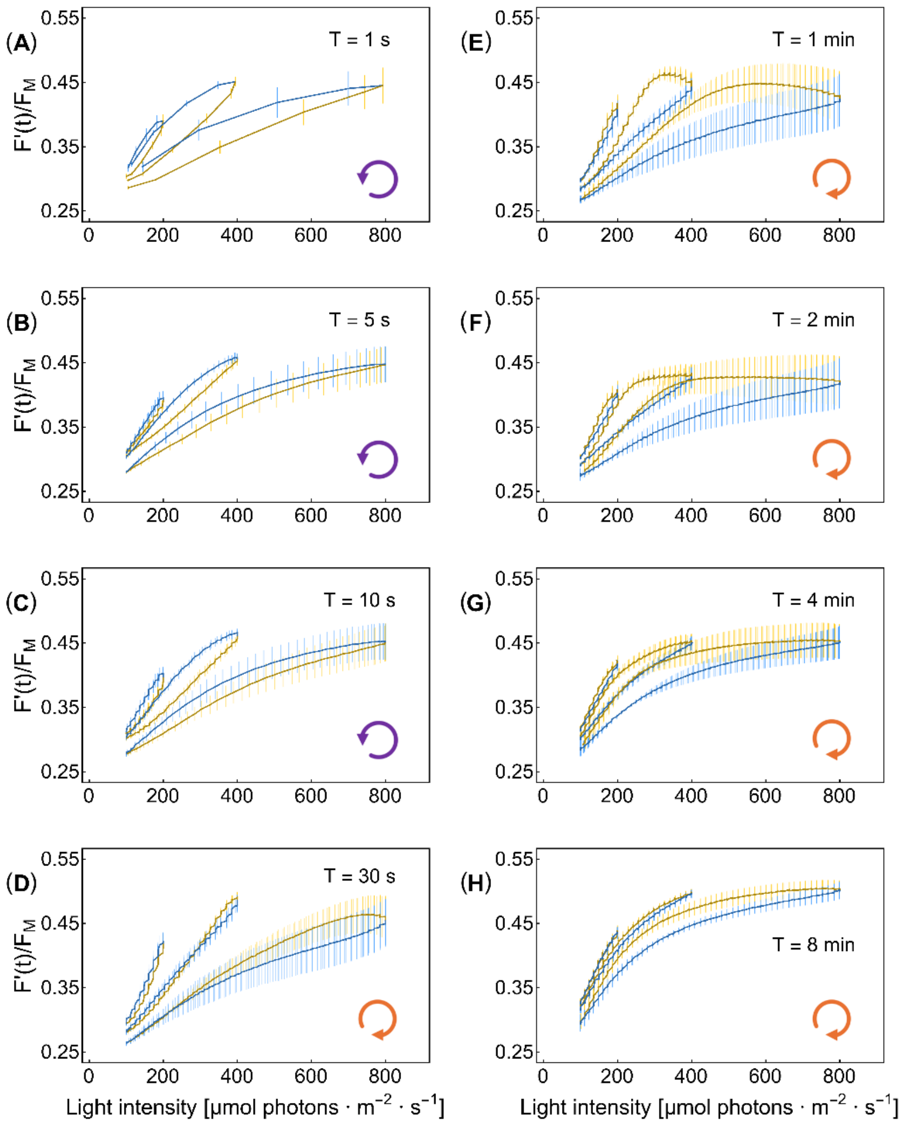

Figure SI-4 The dynamics of the original experimental data on ChlF(t) obtained with the *A. thaliana npq1* mutant. The marking and legends are the same as in Figure SI-1.

The ChlF experimental data on the *npq1* mutant fitted by the four harmonic components of the function in Eq. 1.

The agreement between Figs. SI-4 and SI-5 shows that the numerical fitting did not change the dynamic pattern.

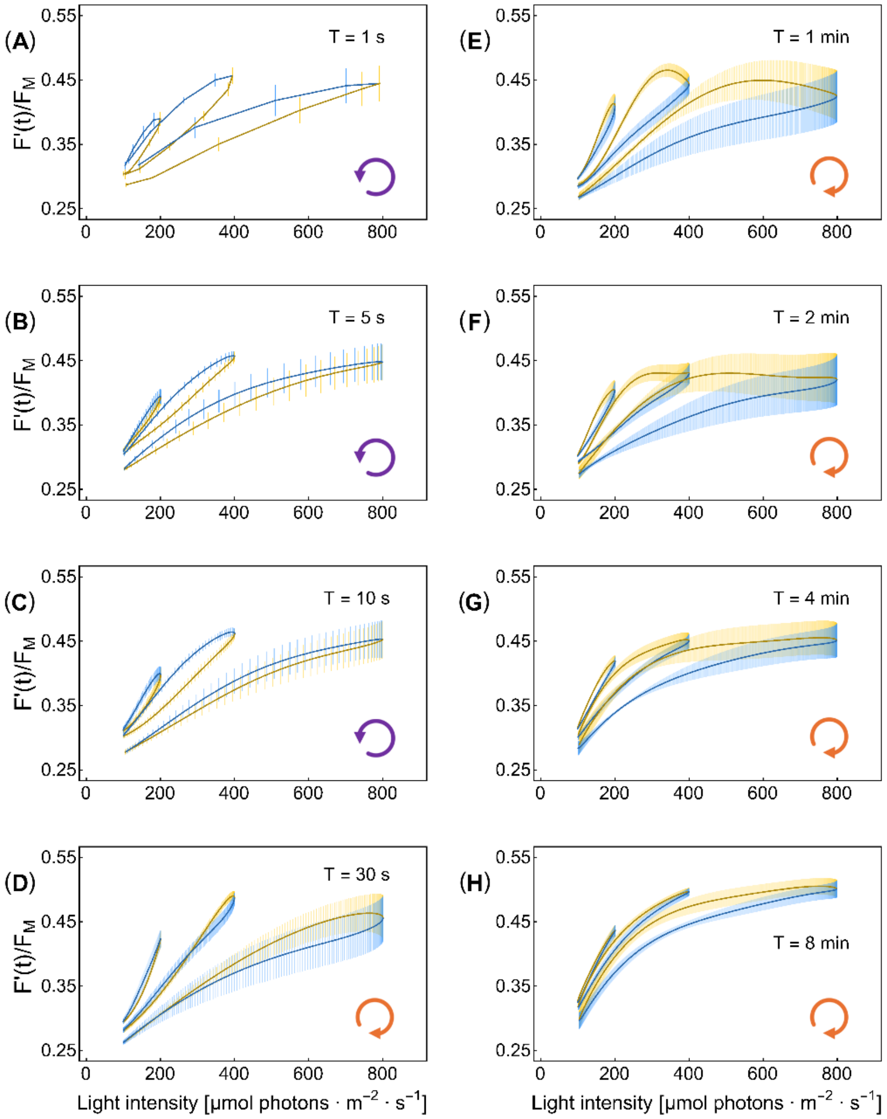

Figure SI-5 Data on *A. thaliana npq1* mutant represented in Figure SI-4 fitted by the function in Eq. 1, the fits averaged and analyzed for experimental error and represented here by resulting analytical function.

Model simulations of the non-quenching states and of the reduced

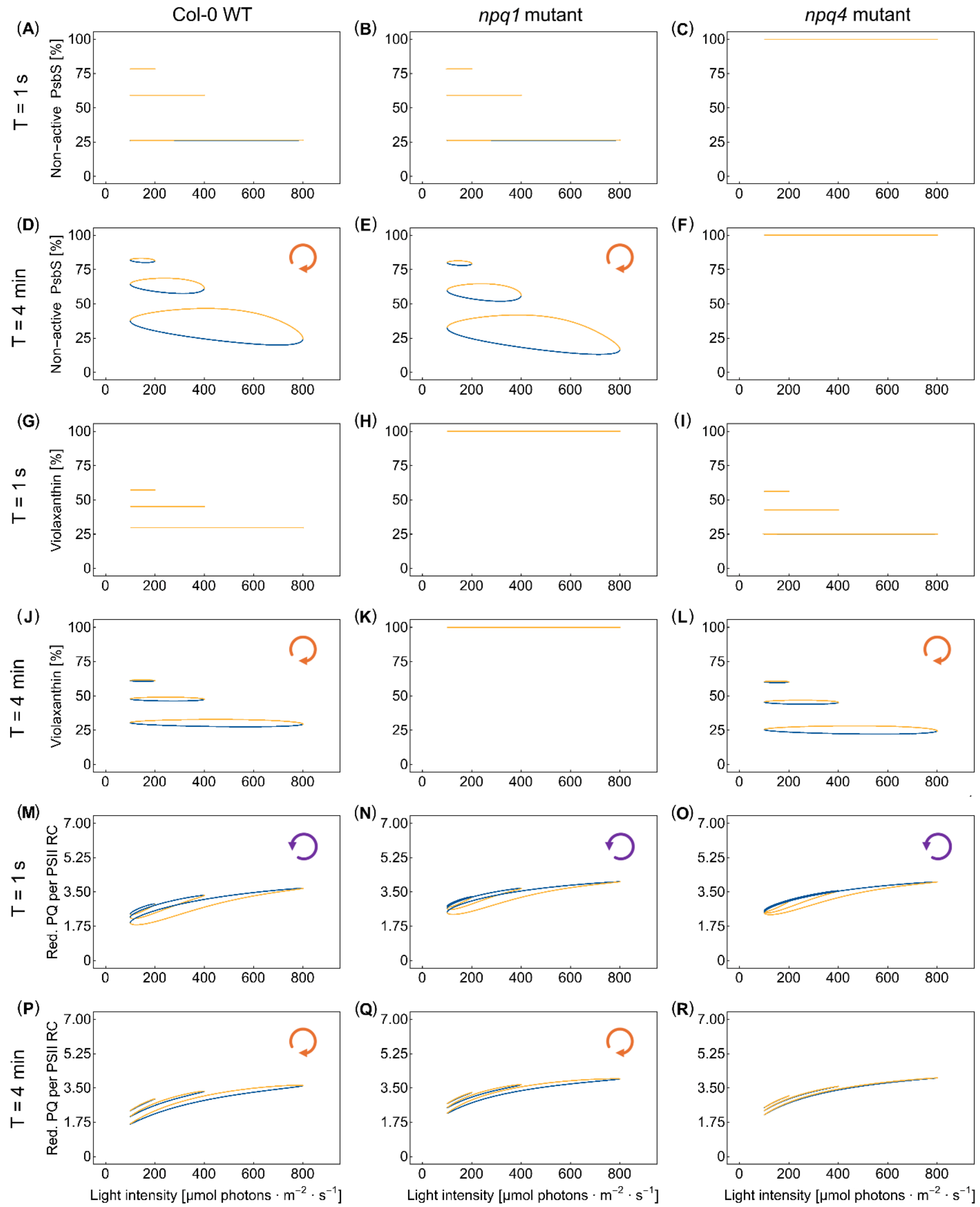

Figure SI-6 The simulated dynamics of non-active PsbS (= 1 - $PsbS_{act}(t)$, panels A - F), of violaxanthin (= 1 - Zea(t), panels G - L) and of reduced PQ pool per PSII reaction center (= PQ_{tot} - PQ(t), panels M - R) of the WT (left column), *npq1* (middle column) and *npq4* (right column) mutants. The period of the oscillating light (T = 1 s or 4 min) is indicated in the legend on the left. The color code and symbols are the same as in the figures in the main text.

References in the Supporting information

Fuente D, Orlando M, Bailleul B, Jullien L, Lazár D, Nedbal L. 2024. A mathematical model to simulate the dynamics of photosynthetic light reactions under harmonically oscillating light. *Plant Physiology and Biochemistry* **217**: 109138.

Lazár D, Nauš J, 1998. Statistical properties of chlorophyll fluorescence induction parameters. *Photosynthetica* **35**(1): 121-127.

Oxborough K, Baker NR. 1997. Resolving chlorophyll *a* fluorescence images of photosynthetic efficiency into photochemical and non-photochemical components – calculation of qP and F_V'/F_M' without measuring F_0'. *Photosynthesis Research* **54**(2): 135-142
